# On the Physical Layer Security Characteristics for MIMO-SVD Techniques for SC-FDE Schemes

**DOI:** 10.3390/s19214757

**Published:** 2019-11-01

**Authors:** João Madeira, João Guerreiro, Rui Dinis, Paulo Montezuma, Luís Miguel Campos

**Affiliations:** 1 FCT, Universidade Nova de Lisboa, Monte de Caparica, 2829-516 Caparica, Portugal; rdinis@fct.unl.pt (R.D.); pmc@fct.unl.pt (P.M.); 2 UAL, Universidade Autónoma de Lisboa, 1169-023 Lisboa, Portugal; jfguerreiro@autonoma.pt; 3 IT, Instituto de Telecomunicações, 1049-001 Lisboa, Portugal; 4 PDMFC, Projecto Desenvolvimento Manutenção Formação e Consultadoria LDA, 1300-609 Lisboa, Portugal; luis.campos@pdmfc.com

**Keywords:** MIMO, SC-FDE, Physical Layer Security, SVD

## Abstract

Multi-Input, Multi-Output (MIMO) techniques are seeing widespread usage in wireless communication systems due to their large capacity gains. On the other hand, security is a concern of any wireless system, which can make schemes that implement physical layer security key in assuring secure communications. In this paper, we study the physical layer security issues of MIMO with Singular Value Decomposition (SVD) schemes, employed along with Single-Carrier with Frequency-Domain Equalization (SC-FDE) techniques. More concretely. the security potential against an unintended eavesdropper is analysed, and it is shown that the higher the distance between the eavesdropper and the transmitter or receiver, the higher the secrecy rate. In addition, in a scenario where there is Line of Sight (LOS) between all users, it is shown that the secrecy rate can be even higher than in the previous scenario. Therefore, MIMO-SVD schemes combined with SC-FDE can be an efficient option for highly secure MIMO communications.

## 1. Introduction

Multiple-Input, Multiple-Output (MIMO) techniques are being increasingly considered for new wireless communication systems, due to their huge capacity over traditional single-antenna techniques. In fact, it can be shown that the capacity can even scale linearly with the number of antenna elements [1,2,3,4]. As such, several MIMO techniques have been selected to integrate wireless communications standards, such as WiFi [5] and Long Term Evolution (LTE) [6], and will likely be key elements in future 5G systems [7].

Although wireless channels have considerable advantages, they also present additional security difficulties when compared with wired channels. In fact, since anyone in range can listen to the channel (such as an eavesdropper that knows the transmitting characteristics such as the frame and block structures and carrier frequency), the security levels of conventional wired communications might not be enough, particularly for Internet-of-Things (IoT) devices [8]. Therefore, it is desirable to have an additional physical layer security level [9,10,11] on top of conventional security measures, so as to increase the overall system security. Thanks to their increased security capabilities, the physical layer security techniques have become increasingly attractive for both industry, [12] and IoT applications [13]. Security measures in the physical layer can take advantage of the different characteristics of the legitimate and eavesdropper links, which can be done using channel estimates, equalization schemes, beamforming, randomised cyclic prefix (CP), among others [14,15,16]. There is significant research of this subject for OFDM systems [17,18], however, there are few published works on this subject for Single Carrier with Frequency Domain Equalization (SC-FDE) systems.

Recently, a promising MIMO technique for SC-FDE was proposed in [19]. This technique employs a Singular Value Decomposition (SVD) scheme [20,21] that combines precoding [22] and decoding [23] at the frequency level, along with a powerful receiver based on the Iterative Block Decision Feedback Equalizer (IB-DFE) [24], that allows for excellent performance, even in severely time-dispersive channels. Techniques based on SVD, such as this one, can be an interesting option for 5G systems [25,26].

In this paper, we consider MIMO-SVD schemes combined with SC-FDE techniques as in [19]. By taking advantage of the different legitimate and eavesdropper’s channels, we analyze the potential security at the physical layer. It is shown that the secrecy rate increases sharply with the distance between the eavesdropper and the transmitter or the receiver, which means that we can have highly secure MIMO communications whenever the eavesdropper is not co-located with the transmitter or the receiver, even if the eavesdropper is able to receive all the training blocks shared between the legitimate transmitter and receiver.

The notation is as follows: bold letters denote matrices or vectors. Capital letters are associated to the frequency-domain and small letters are associated to the time-domain. ${\left(\cdot \right)}^{H}$ denotes the Hermitian operator. $I_{P}$ denotes a P×P identity matrix. E[·] represent the expected value.

This paper is organized as follows: in Section 2 we begin by characterizing the point-to-point MIMO system with its intended receiver B and eavesdropper E, followed by an analysis of the system capacity and secrecy rate calculations. Section 3 shows the simulated Bit Error Rate (BER) and secrecy rate for both transmitter-receiver pairs. In addition, results and corresponding analysis are presented for a scenario where there is a Line-of-Sight (LOS) link between all users. Lastly, Section 4 concludes this paper.

## 2. Materials and Methods

### 2.1. System Characterization

**Figure 1:**
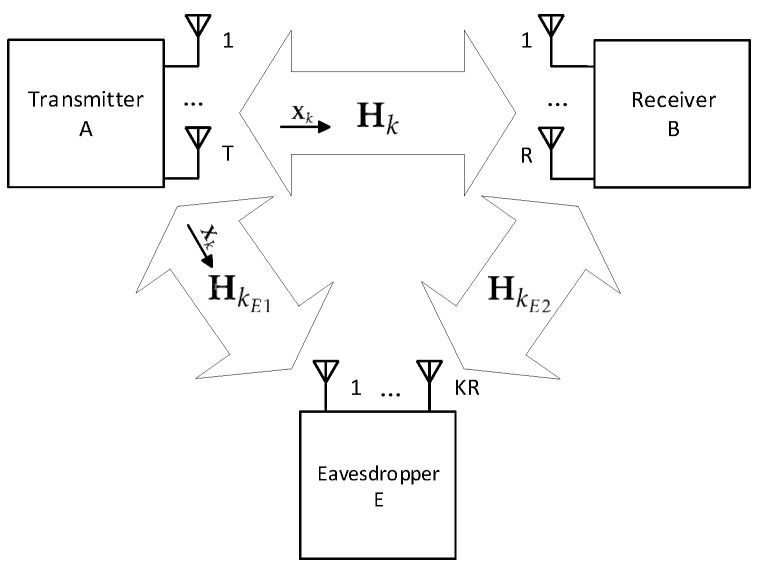
Considered MIMO-SVD system with an eavesdropper.

**Figure 2:**
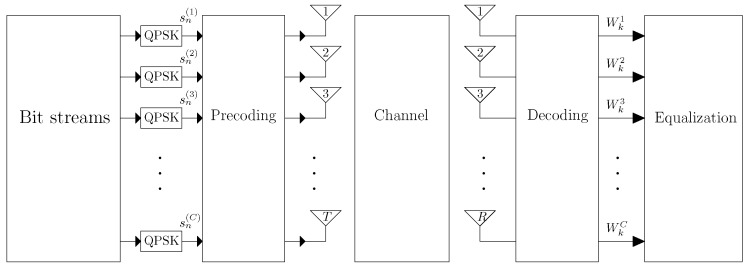
Proposed MIMO SVD-based system, employing *T* transmitting antennas and *R* receiving antennas.

In this paper we consider a point-to-point MIMO system with a transmitter, denoted A (Alice in conventional wiretap channels nomenclature), employing *T* antennas and a receiver, denoted B (Bob in conventional wiretap channels nomenclature), employing *R* antennas. For the sake of simplicity we assume T=R, although this work could easily be extended to the case where T≠R. In addition, there is a third user, denoted E (Eve in conventional wiretap channels nomenclature), that is attempting to eavesdrop the signal transmitted between A and B. Although we are assuming a scenario with a single eavesdropper, a scenario with more eavesdroppers can also be taken into account [27]. However, a scenario with multiple co-located eavesdroppers, can be approximated by a single eavesdropper with KR antennas, where *K* is the number of eavesdroppers. This three user scenario is shown in Figure 1. The distance between each antenna at the transmitter and the receiver is assumed much larger than the transmitted signal’s wavelength, and the receiver is in the far field region of the transmitter. The transmitter can send up to C=R data streams over a highly frequency-selective channel. To cope with the strong levels of inter-symbol interference (ISI) associated to such channels, we employ an SC-FDE transmission technique. The data blocks are composed of *N* quadrature phase shift keying (QPSK) symbols (the generalization to other constellations with IB-DFE is straightforward [28]), plus an appropriate CP that is larger than the maximum overall channel impulse response. A block diagram of the considered system is depicted in Figure 2.

The data symbols to be transmitted by the *C* single-carrier data streams will be denoted by the N×C matrix s, where each data stream is represented as an N×1 vector $s^{\left(c\right)}=\left[s_{1}^{\left(c\right)}\;s_{2}^{\left(c\right)}\;\cdots \;s_{N}^{\left(c\right)}\right]$. In that context, $s_{n}^{\left(c\right)}$ represents the QPSK symbol transmitted on the *c*th stream at the *n*th time instant. The frequency-domain counterpart of the transmitted data is defined by the discrete Fourier transform (DFT) of s, which is the N×C matrix S. The group of symbols associated to the *k*th sub-carrier are represented as the 1×C vector $S_{k}=\left[S_{k}^{\left(1\right)}\;S_{k}^{\left(2\right)}\;...\;S_{k}^{\left(C\right)}\right]$.

The channel frequency response for the *k*th sub-carrier is modeled by the R×T matrix

(1) $$H_{k}=\left[\begin{array}{cccc} H_{k}^{\left(1,1\right)} & H_{k}^{\left(1,2\right)} & \cdots & H_{k}^{\left(1,T\right)}\\ H_{k}^{\left(2,1\right)} & H_{k}^{\left(2,2\right)} & \cdots & H_{k}^{\left(2,T\right)}\\ \vdots & \vdots & \ddots & \vdots \\ H_{k}^{\left(R,1\right)} & H_{k}^{\left(R,2\right)} & \cdots & H_{k}^{\left(R,T\right)} \end{array}\right].$$

Since we are considering a point-to-point communication where we have a multi-antenna transmitter and a multi-antenna receiver, the separation of the MIMO streams can be done using the SVD technique [20]. To perform the SVD, we need channel knowledge at both the transmitter and receiver. To achieve this, the transmitter and receiver exchange training sequences. This process is relatively straightforward in time division duplex (TDD) schemes, where we can take advantage of the channel’s reciprocity.

The SVD technique allows us to obtain up to *C* decoupled channels, onto which we can multiplex up to *C* data streams. Since we are employing SC-FDE schemes over frequency-selective channels, this decomposition is made at the sub-carrier level. Therefore, we can decompose the channel matrix associated to a given sub-carrier $H_{k}$ as

(2) $$H_{k}=U_{k}\Lambda_{k}V_{k}^{H},$$

where $U_{k}$ is the R×R decoding matrix, $V_{k}$ is the T×T precoding matrix and $\Lambda_{k}$ is a C×C diagonal matrix composed by the singular values of $H_{k}$, which are sorted in descending order according to their power.

#### 2.1.1. Transmission

Although SVD techniques allow for the orthogonalisation of the different data streams, the performance associated to each steam can vary substantially. This is explained by the fact that the performance depends essentially on the magnitude of the singular values, which vary considerably from stream to stream [29]. To overcome this problem, one can employ appropriate loading techniques, with power and/or constellation differentiation between different streams, as it is proposed for some OFDM-based systems [30]. An interesting alternative for SC-FDE MIMO-SVD systems was described in [19], which is based on interleaving the data to be transmitted between all streams, thereby forcing each stream to be affected by singular values with different powers, and avoiding streams with very poor performance (that would determine the average BER performance). We define $S_{k}^{{}^{\prime }}$ as the interleaved data symbols associated with sub-carrier.

As already pointed out, the channel estimates at the transmitter side, required for computing the precoding matrix, can be obtained from a training sequence that was previously sent by the receiver. After that, the transmitter sends a training sequence to the receiver (typically at the beginning of the data block), which is used by the receiver to compute the detection matrix, perform the channel equalization and complete the SVD decomposition (the details are described below). Naturally, we assume that the channel coherence time is greater than the time it takes to transmit both sequences and the data block. The eavesdropper listens to both training sequences, so it can compute its own channel estimate.

**Figure 3:**
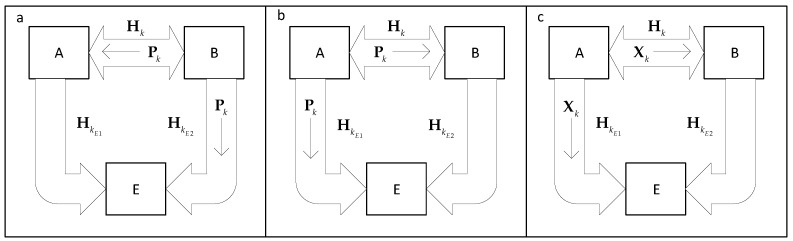
Steps for obtaining the channel estimates. (**a**) the receiver sends a training sequence, $P_{k}$, that is received by the transmitter and the eavesdropper. (**b**) the transmitter sends a training sequence that is received by the receiver and the eavesdropper. (**c**) the transmitter begins sending data to the receiver, that is also received by the eavesdropper.

We can summarize this process into three steps, as shown in Figure 3. In the first step, the receiver sends a training sequence to the transmitter, which is overheard by the eavesdropper. In this step, both the transmitter and eavesdropper obtain channel estimates. In the second step, the transmitter sends a sequence of training symbols, so that the receiver can obtain a channel estimate and compute the decoding matrix to complete the SVD. The eavesdropper also listens to this sequence and obtains another channel estimate. The third and last step is when the data transmission begins. The transmitter uses its channel estimate to precode the signal, while the receiver uses its channel estimate in order to perform the decoding of the received signal. Similarly, the eavesdropper tries to decode the overheard signal. To increase the accuracy of its detection, the eavesdropper uses a channel calculated as an average of its two channel estimates.

As described in [31], the channel can be expressed as

(3) $$H_{k}=\rho_{A}{\hat{H}}_{k_{A}}+\epsilon_{k},$$

where ${\hat{H}}_{k_{A}}$ is the channel estimate used by the transmitter, $\rho_{A}$ is a correlation factor with the true channel, and $\epsilon_{k}$ is the error associated to the channel estimation process (our analysis can be easily extended to other models for the channel estimation errors). This error $\epsilon_{k}$ is characterized as a complex variable with Gaussian distribution and variance $2\sigma_{N}^{2}/\beta$, where $\sigma_{N}^{2}$ is the noise variance for a specific Signal-to-Noise Ratio (SNR) value and β is a scaling factor. For β→∞ and $\rho_{A}=1$, there is a perfect channel estimation, i.e., ${\hat{H}}_{k}=H_{k_{A}}$.

The SVD decomposition of ${\hat{H}}_{k_{A}}$ is as follows

(4) $${\hat{H}}_{k_{A}}={\hat{U}}_{k_{A}}{\hat{\Lambda }}_{k_{A}}{\hat{V}}_{k_{A}}^{H}.$$

Therefore, the transmitter computes the precoded symbols with the T×1 vector ${\hat{V}}_{k_{A}}$ as

(5) $$X_{k}={\hat{V}}_{k_{A}}S_{k}^{{}^{\prime }}.$$

#### 2.1.2. Reception

Both the correct receiver and the eavesdropper employ the same reception approach. However, the channel that they observe will be different, i.e., they will work with different channel estimates, since in general the eavesdropper is at a position different from the transmitter and the receiver. We also consider the pessimistic scenario where the eavesdropper knows the interleaving pattern in use (in practice, this could add an extra security layer, that is not considered in this paper).

The received signal can be expressed as

(6) $$Z_{k}=H_{k}X_{k}+N_{k},$$

where $N_{k}$ denotes the frequency-domain additive white Gaussian noise (AWGN) samples associated to the *k*th sub-carrier.

Naturally, both the receiver and the eavesdropper must perform the decoding operation. For the intended receiver, B, we can define the channel as in (3), namely

(7) $$H_{k}=\rho_{B}{\hat{H}}_{k_{B}}+\epsilon_{k}.$$

We can assume that there is little difference between the estimation of the transmitter and the intended receiver, so it is reasonable to approximate $\rho_{A}=\rho_{B}\approx 1$. For the sake of simplicity, we will also assume that the power of the channel estimation error is equal for A and B (the generalization for other cases is straightforward).

The SVD decomposition done at the intended receiver’s side is

(8) $${\hat{H}}_{k_{B}}={\hat{U}}_{k_{B}}{\hat{\Lambda }}_{k_{B}}{\hat{V}}_{k_{B}}^{H},$$

with ${\hat{U}}_{k_{B}}$, ${\hat{\Lambda }}_{k_{B}}$ and ${\hat{V}}_{k_{B}}^{H}$ being the corresponding estimates of the matrices defined in (2). It should be noted that the eavesdropper cannot directly estimate the channel between A and B, since its actual value is never transmitted between A and B. Therefore, the eavesdropper can only approximate such estimation by estimating the channel between A and E and between B and E. The eavesdropper obtains these estimates by listening to the training sequences exchanged between A and B. We can define both of these channels as

(9) $$H_{k_{E1}}=\rho_{E1}{\hat{H}}_{k_{E1}}+\xi_{k}+\epsilon_{k}$$

and

(10) $$H_{k_{E2}}=\rho_{E2}{\hat{H}}_{k_{E2}}+\xi_{k}+\epsilon_{k},$$

with $\rho_{E1}$ and $\rho_{E2}$ referring to the correlation between the different channels and the real channels and $\xi_{k}$ being an appropriate Gaussian distributed error term with variance $\sigma_{N}^{2}/\beta_{M}$, where $\beta_{M}$ is a scaling factor. Since the eavesdropper does not know the channel, we can assume that $\rho_{E1}=\rho_{E2}<1$. As mentioned before, in order to improve the quality of the estimation of the channel between A and B, the eavesdropper can calculate an average of the estimates of the intermediate channels, i.e.,

(11) $$H_{k}=\frac{H_{k_{E1}}+H_{k_{E2}}}{2}.$$

As in conventional SVD techniques, the decoding is made by multiplying the received signal by the decoding matrix estimate ${\hat{U}}_{k_{B}}^{H}$ for the intended receiver; or ${\hat{U}}_{k_{E}}^{H}$ for the eavesdropper. Since the process is the same for both receivers, we will use ${\hat{U}}_{k}^{H}$ as a place-holder for either receiver. The decoding is then computed as

(12) $$W_{k}^{\prime }={\hat{U}}_{k}^{H}Z_{k},$$

where $W_{k}^{\prime }$ is a C×1 column vector with the interleaved, decoded symbols. These symbols can be written as

(13) $$\begin{array}{c} W_{k}^{\prime }={\hat{\Lambda }}_{k}S_{k}^{\prime }+{\hat{U}}_{k}^{H}N_{k}, \end{array}$$

with ${\hat{\Lambda }}_{k}$ corresponding to the diagonal matrix composed by the singular values of the estimated channel.

However, before performing equalization, we must group all the data symbols associated to a given stream, i.e., restore the original symbol order. This is done by applying the deinterleaving to the matrix $W_{k}^{\prime }$, which yields

(14) $$W_{k}={\hat{\Lambda }}_{k}^{\prime }S_{k}+{\hat{U}}_{k}^{\prime H}N_{k}^{\prime }.$$

Thanks to the interleaving, each stream becomes affected by a frequency-selective channel, composed by the interleaving of the different singular values.

#### 2.1.3. Multiple Eavesdroppers

Let us now assume a scenario with K eavesdroppers. Moreover, let us consider the worst case, i.e., the case where the different eavesdroppers are co-located and can perform joint estimation and equalisation. Under these conditions, we can model the existence of *K* eavesdroppers by considering one eavesdropper with KR receive antennas. Thus, the channel being estimated by the eavesdroppers can be defined as

(15) $$H_{k_{E}}=\frac{H_{k_{E1}}+H_{k_{E2}}}{2}.$$

The received signal $Z_{k_{E}}$ is expressed as

(16) $$Z_{k_{E}}=H_{k_{E}}X_{k}+N_{k}.$$

It should be noted that the eavesdroppers do not require any changes to the equalization algorithm, since the number of singular values is the same, not to mention they can take advantage of the increased singular value power due to employing more receiving antennas. Considering the SVD, the channel represented in (15) can be decomposed as

(17) $${\hat{H}}_{k_{E}}={\hat{U}}_{k_{E}}{\hat{\Lambda }}_{k_{E}}{\hat{V}}_{k_{E}}^{H},$$

where ${\hat{\Lambda }}_{k_{E}}$ is a C×C diagonal matrix composed by the singular values of ${\hat{H}}_{k_{E}}$, ${\hat{V}}_{k_{E}}$ is the T×T precoding matrix, that is not utilised by the eavesdroppers, and ${\hat{U}}_{k_{E}}$ is the KR×T decoding matrix, computed economically so as to not have null columns.

#### 2.1.4. Line-of-Sight Link Scenario

Another possible scenario is the one where there is LOS between the transmitter and both the receiver and eavesdropper. Under these conditions, the channel is defined as the sum of a LOS component (that does not suffer fading effects) with several multipath rays (assumed uncorrelated and with fading effects). In a worst case scenario, we can assume that the eavesdropper can estimate the LOS component (eventually with a certain error), although that is not feasible for the remaining multipath rays [32]. In this case, we define the channel as

(18) $$H_{k,los}=D_{k_{los}}+R_{k_{mp}},$$

where $D_{k_{los}}$ is the low-fading, highly-correlated LOS component and $R_{k_{mp}}$ is the high-fading multipath component of the channel. We then substitute this channel in (3) and (7), as

(19) $$H_{k,los}=\rho_{A}{\hat{H}}_{k_{A},los}+\epsilon_{k},$$

(20) $$H_{k,los}=\rho_{B}{\hat{H}}_{k_{B},los}+\epsilon_{k}.$$

The intended receiver and transmitter’s remaining operations are calculated as described previously.

The eavesdropper, however, cannot estimate the multipath component of the channel, and must instead rely on the estimate of the LOS component. We define this component as

(21) $$D_{k_{los}}=\frac{H_{k_{E1,los}}+H_{k_{E2,los}}}{2},$$

where

(22) $$H_{k_{E1,los}}=\rho_{E1}{\hat{H}}_{k_{E1,los}}+\xi_{k}+\epsilon_{k}$$

and

(23) $$H_{k_{E2,los}}=\rho_{E2}{\hat{H}}_{k_{E2,los}}+\xi_{k}+\epsilon_{k}.$$

In this scenario, the channel estimates ${\hat{H}}_{k_{E1,los}}$ and ${\hat{H}}_{k_{E2,los}}$ only concerns the LOS component between A and E and B and E, respectively. The difference between these estimates and the real channel will be proportional to the power of the multipath component. We define the ray power coefficient as

(24) $$\alpha_{RP}=\frac{P_{R}}{P_{D}+P_{R}},$$

where $P_{D}$ and $P_{R}$ are the powers of the LOS and multipath components, respectively. Clearly, if $\alpha_{RP}=0$, the channel is only composed by the LOS component, whereas at $\alpha_{RP}=1$ the channel is composed of only the multipath component.

#### 2.1.5. Iterative Equalization

In order to reduce the ISI, we employ a nonlinear FDE technique based on the IB-DFE concept [24,33]. The IB-DFE is a frequency-domain receiver which utilizes an iterative equalization based on the minimum mean squared error (MMSE). This equalization is done on a sub-carrier basis, and is composed by a feedforward and feedback equalization, which is employed to remove the residual ISI. The equalization processes are iterative and can be repeated up to *L* times.

The set of equalized symbols associated with the *k*th sub-carrier and *l*th iteration are given by

(25) $${\tilde{S}}_{k}^{\left(l\right)}=F_{k}^{\left(l\right)}W_{k}-B_{k}^{\left(l\right)}{\bar{S}}_{k}^{\left(l-1\right)},$$

where ${\bar{S}}_{k}$ is a C×1 matrix with the soft-decisions of the previous iteration, and $F_{k}^{\left(l\right)}$ and $B_{k}^{\left(l\right)}$ are the feedforward and feedback equalization matrices for the *k*th sub-carrier and *l*th iteration, respectively. The feedforward equalization matrix for the *k*th sub-carrier and *l*th iteration is defined as

(26) $$F_{k}^{\left(l\right)}=\frac{{\hat{\Lambda }}_{k}^{{}^{\prime }}}{\left(1-{\left|\rho^{\left(l-1\right)}\right|}^{2}\right){\hat{\Lambda }}_{k}^{{}^{\prime }2}+\frac{1}{\mathrm{SNR}}},$$

where $\rho^{\left(l-1\right)}$ denotes the block-wise reliability associated to the data estimated in the (l−1)th iteration (when l=1, we have $\rho^{\left(0\right)}=0$). On the other hand, the feedback equalization matrix is defined as

(27) $$B_{k}^{\left(l\right)}=F_{k}^{\left(l\right)}{\hat{\Lambda }}_{k}^{{}^{\prime }}-I.$$

The soft-decision estimates of the transmitted data, employed in the feedback equalization, are calculated using the reliability of each bit in each symbol, expressed as a log-likelihood ratio (LLR), as

(28) Ln(I,i)=2σi2Res˜n(i),

and

(29) Ln(Q,i)=2σi2Ims˜n(i),

where

(30) $$\sigma_{i}^{2}=\frac{1}{2}E\left[{\left|s_{n}-{\tilde{s}}_{n}^{\left(i\right)}\right|}^{2}\right]\approx \frac{1}{2N}\sum_{n=0}^{N-1}{\left|{\hat{s}}_{n}-{\tilde{s}}_{n}^{\left(i\right)}\right|}^{2}.$$

After obtaining the LLR for each bit, we can calculate the soft decision of a given data symbol as

(31) $${\bar{s}}_{n}^{\left(i\right)}=\tanh\left(\frac{L_{n}^{\left(I,i\right)}}{2}\right)+j\tanh\left(\frac{L_{n}^{\left(Q,i\right)}}{2}\right).$$

The estimated data symbols are obtained through the hard-decision of the equalized symbols.

### 2.2. Secrecy Rate

The secrecy rate is defined as the difference between the capacity of the channel between A and B, and the capacity of the channel between A and E [34,35,36]. For simplicity, we define the total capacity as the sum of the capacity of each sub-carrier, i.e.,

(32) $$C=\sum_{k=1}^{N}C_{k},$$

where $C_{k}$ denotes the capacity of a single sub-carrier, defined as [3]

(33) $$C_{k}=I\left(X_{k},Z_{k}\right),$$

where $I(X_{k},Z_{k})$ is the mutual information between the transmitted signal and the received signal, which can be computed by

(34) $$I\left(X_{k},Z_{k}\right)=H\left(Z_{k}\right)-H(Z_{k}\left|X_{k}\right),$$

with $H(Z_{k})$ being the differential entropy of $Z_{k}$ and $H(Z_{k}\left|X_{k}\right)$ being the conditional differential entropy of $Z_{k}$ given $X_{k}$. Since we know that $X_{k}$ is independent from $N_{k}$, we can simplify $H(Z_{k}\left|X_{k}\right)=H\left(N_{k}\right)$ and define both entropies as

(35) $$H\left(Z_{k}\right)=\log_{2}\left({\left(\pi e\right)}^{C}\det\left(H_{k}R_{X}H_{k}^{H}+R_{N}\right)\right),$$

and

(36) $$H\left(N_{k}\right)=\log_{2}\left({\left(\pi e\right)}^{C}\det\left(R_{N}\right)\right),$$

where $R_{X}=\sigma_{X}^{2}I$ and $R_{N}=\sigma_{N}^{2}I$, with $\sigma_{X}^{2}$ and $\sigma_{N}^{2}$ corresponding to the variances of $X_{k}$ and $N_{k}$, respectively. By substituting (35) and (36) in (34), we can write

(37) $$\begin{array}{c} I\left(X_{k},Z_{k}\right)=\sum_{c=1}^{C}\log_{2}\left(1+{\left|\lambda_{c}\right|}^{2}\mathrm{SNR}\right). \end{array}$$

Since we have two different transmitter-receiver pairs, we can, likewise, define two different system capacities. Let us start by defining the system capacity associated with the link from A to B (i.e., the capacity of the intended receiver), which is given by

(38) $$C_{k}^{AB}=\sum_{c=1}^{C}\log_{2}\left(1+{\left|\lambda_{c}\rho_{B}\right|}^{2}\frac{\sigma_{X}^{2}}{\sigma_{N}^{2}+\sigma_{B}^{2}}\right),$$

where $\sigma_{B}^{2}$ is the power of the interference associated with the imperfect channel estimation, given by

(39) $$2\sigma_{B}^{2}=E\left[{\hat{\Lambda }}_{k_{B}}^{I}{\hat{\Lambda }}_{k_{B}}^{IH}\right],$$

with ${\hat{\Lambda }}_{k_{B}}^{I}$ denoting a matrix comprised of the interference in the receiver, which can be computed as

(40) $${\hat{\Lambda }}_{k_{B}}^{I}={\hat{\Lambda }}_{k_{B}}-\mathrm{diag}\left({\hat{\Lambda }}_{k_{B}}\right).$$

Similarly, we can define the capacity of the eavesdropper as

(41) $$C_{k}^{AE}=\sum_{c=1}^{C}\log_{2}\left(1+{\left|\lambda_{c_{E}}\rho_{E}\right|}^{2}\frac{\sigma_{X}^{2}}{\sigma_{N}^{2}+\sigma_{E}^{2}}\right),$$

where $\rho_{E}$ is a simplification defined as $\rho_{E}=\rho_{E1}=\rho_{E2}$, and $\sigma_{E}^{2}$ is the interference power due to the imperfect channel estimation, which is larger than $\sigma_{B}^{2}$, and is computed as

(42) $$2\sigma_{E}^{2}=E\left[{\hat{\Lambda }}_{k_{E}}^{I}{\hat{\Lambda }}_{k_{E}}^{IH}\right].$$

Likewise, ${\hat{\Lambda }}_{k_{E}}^{I}$ is the interference matrix computed as

(43) $${\hat{\Lambda }}_{k_{E}}^{I}={\hat{\Lambda }}_{k_{E}}-\mathrm{diag}\left({\hat{\Lambda }}_{k_{E}}\right).$$

With (38) and (41), we are able to obtain the total capacity by using (32). Moreover, we are also able to compute the secrecy rate, defined by the difference between the intended receiver’s capacity and the eavesdropper’s capacity, i.e.,

(44) $$SR=C^{AB}-C^{AE}.$$

## 3. Results and Discussion

In this section we present a set of performance results regarding the BER and secrecy rate of the considered point-to-point MIMO system with, unless otherwise mentioned, T=8 transmit antennas and R=8 receive antennas. These performance results involve scenarios with and without a LOS component and are obtained through Monte Carlo simulations. Unless otherwise stated, the block size is N=256.

### 3.1. NLOS Scenario

**Figure 4:**
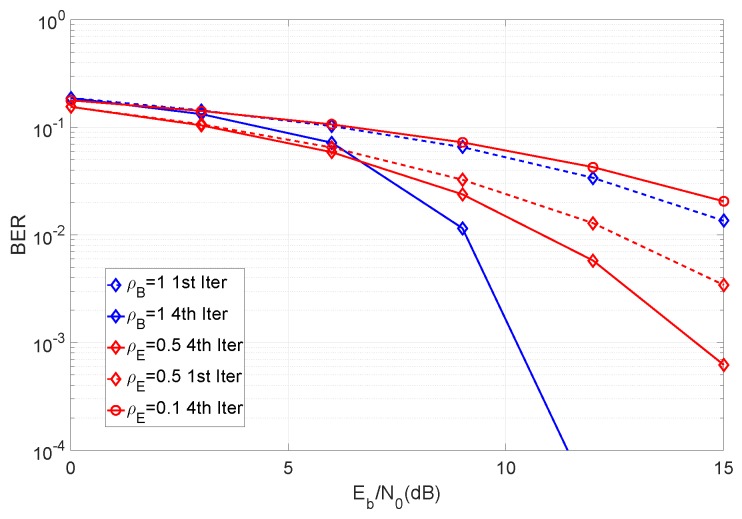
Comparison of BER for different values of $\rho_{E}$, with $\beta_{N}=\beta_{M}=\infty$.

We begin by analyzing the impact of the $\rho_{E}$ factor in the receiver’s performance. This can be observed in Figure 4, where we measure BER of the eavesdropper for different $\rho_{E}$ values.

**Figure 5:**
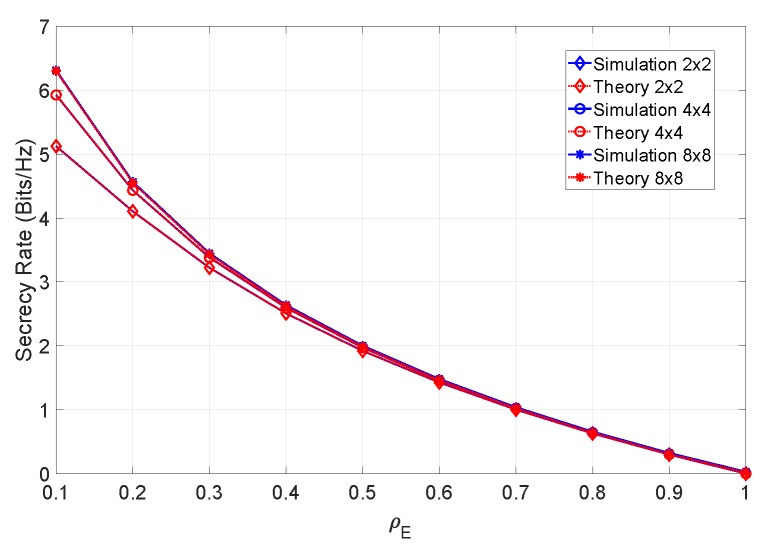
Secrecy rate of the system for an SNR of 12 dB and different MIMO configurations.

From the figure, it can be observed that the system performance can be severely degraded at low levels of $\rho_{E}$. In accordance with our system definition, it is not unreasonable to assume that the eavesdropper will operate with small values of $\rho_{E}$. In the next set of results, we compute the secrecy rate of the system under different conditions. Figure 5 shows the secrecy rate as a function of $\rho_{E}$, considering different MIMO configurations.

**Figure 6:**
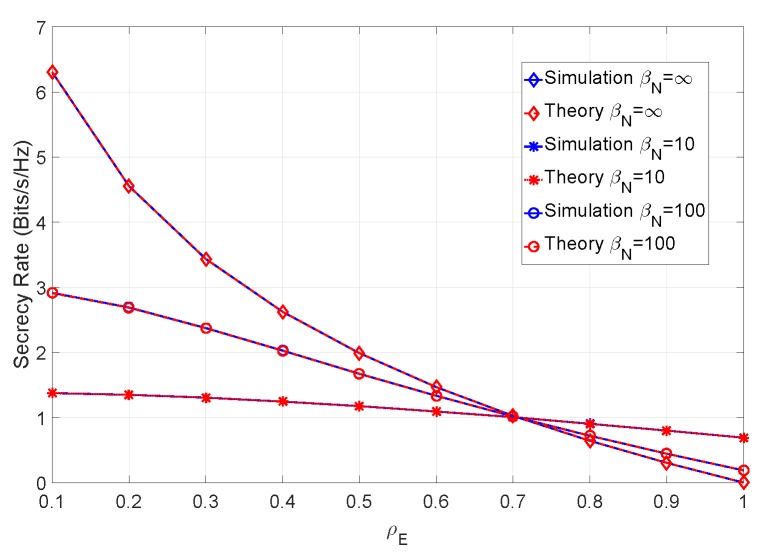
Secrecy rate of the system for an SNR of 12 dB and various levels of channel estimation error on both sides.

From the figure it can be concluded that, with perfect CSI, the maximum attainable secrecy rate increases with the number of antennas of both users. Figure 6 shows the secrecy rate of an 8×8 system, considering different values of $\beta_{N}$ (i.e., considering different channel estimation errors on both receivers), at an SNR of 12 dB.

**Figure 7:**
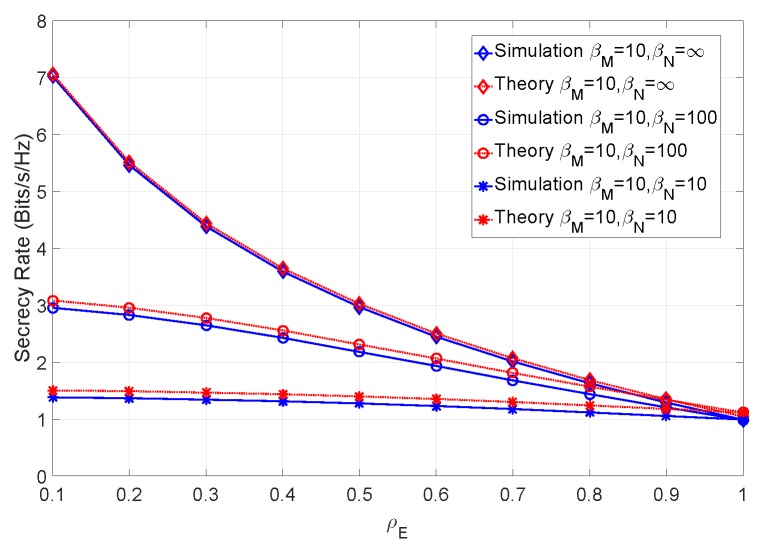
Secrecy rate of the system for an SNR of 12 dB and $\beta_{M}=10$ with various levels of channel estimation error.

As expected, the addition of a channel estimation error negatively impacts the secrecy rate of the system, particularly for lower values of $\rho_{E}$. In Figure 7, we have introduced the channel mismatch error, represented by $\beta_{M}$, in addition to the channel estimation error and SNR of the previous simulations.

**Figure 8:**
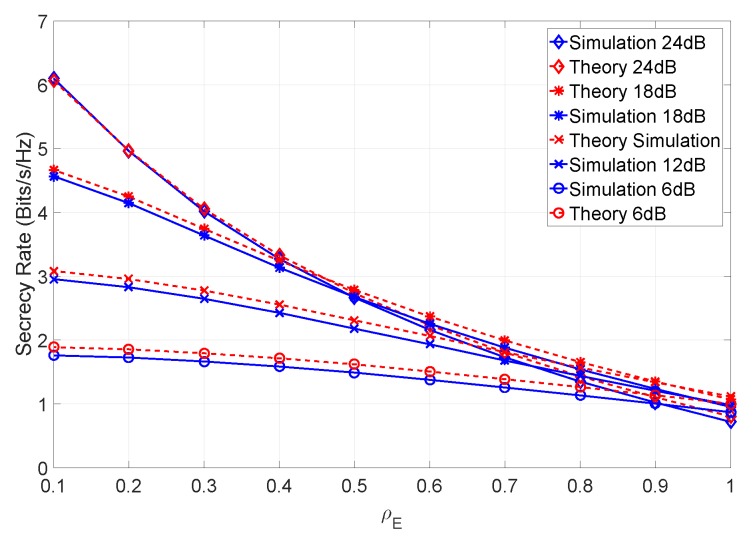
Secrecy rate of the system for different levels of SNR and with $\beta_{N}=100$ and $\beta_{M}=10$.

From the figure, it can be seen that when the channel estimation error assumes low levels, the secrecy rate increases. It should also be noted that even for high values of $\rho_{E}$, the secrecy rate is higher than in a scenario with no channel mismatch error. This is expected due to the mismatch error affecting only the eavesdropper’s capacity. In addition, a relatively small difference between the theoretical and simulated results can be observed. This arises due to the residual error of the Gaussian approximation. Figure 8 combines various levels of SNR for the same levels of channel estimation and mismatch errors.

From the figure, it can be noted that a higher SNR leads to a higher secrecy rate, as expected, with the secrecy rate gain increasing further for smaller values of $\rho_{E}$.

### 3.2. Multiple Eavesdroppers Scenario

**Figure 9:**
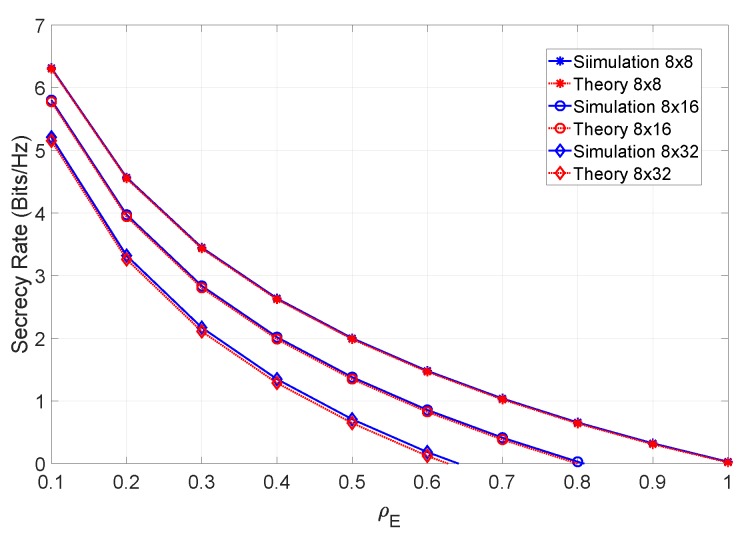
Secrecy rate of the system for various numbers of eavesdroppers, at 12 dB SNR.

Let us now consider the existence of *K* eavesdroppers performing joint estimation and equalization. As mentioned before, this scenario is approximated by a single eavesdropper employing KR antennas, for K>1. Figure 9 shows the secrecy rate of the system for K=1,2 and 4.

**Figure 10:**
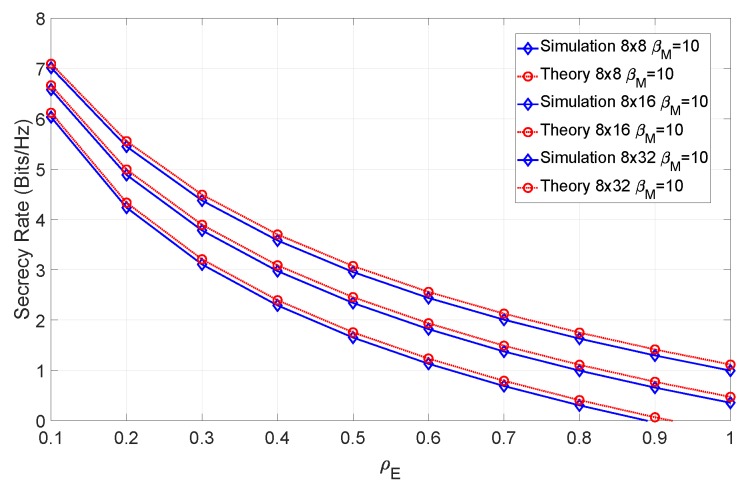
Secrecy rate of the system for various numbers of eavesdroppers and $\beta_{M}=10$, at 12 dB SNR.

From the figure, it can be seen that increasing the number of eavesdroppers leads to a lower attainable secrecy rate. This fact is not limited to the scenario without errors, as can be observed in the scenario with channel mismatch in Figure 10.

From this figure, it can be noted that by considering more eavesdroppers and/or antennas, the impact of the channel mismatch error can be reduced (or even eliminated).

### 3.3. LOS Scenario

**Figure 11:**
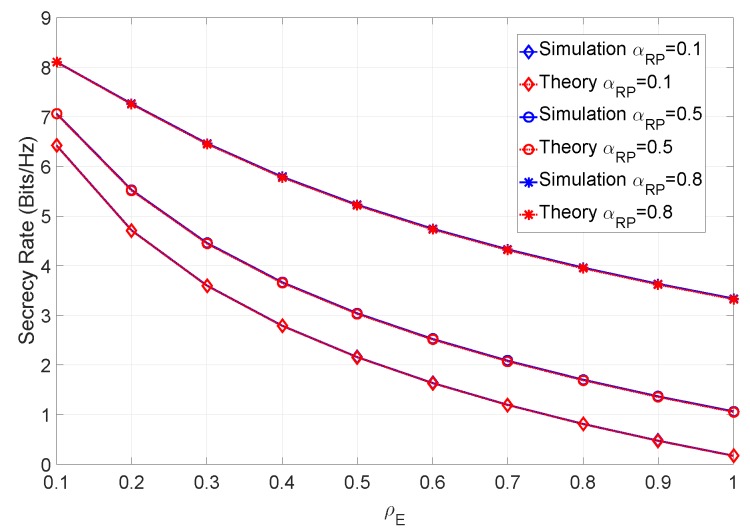
Secrecy rate of the system for various ray power ratios with $\beta_{N}=\infty$.

In addition to varying $\rho_{E}$ and the error factors, let us evaluate the secrecy rate of a scenario where we also vary the ray power ratio between multipath component and main LOS component. Figure 11 shows the secrecy rate with no errors, considering different values of $\rho_{E}$ and different ray power coefficients $\alpha_{RP}$.

**Figure 12:**
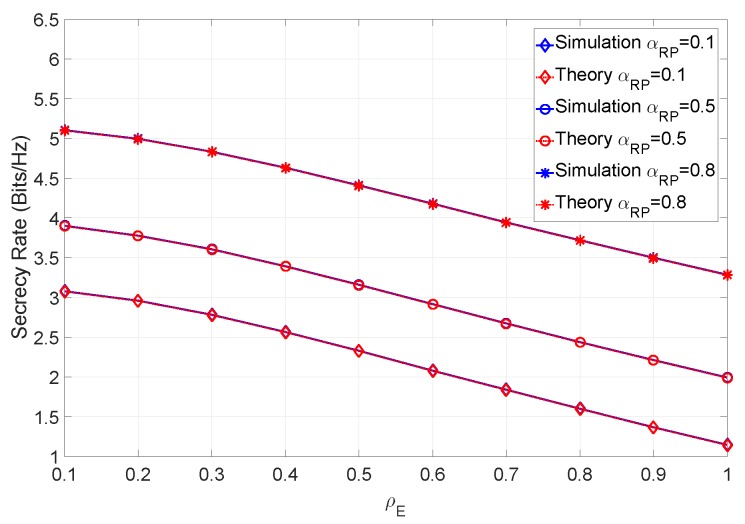
Secrecy rate of the system for various ray power ratios with $\beta_{N}=100$ at 12 dB SNR.

From the figure it can be observed that the higher the ray power ratio, the higher the achievable secrecy rate. In fact, this is somewhat expected, since the component that the eavesdropper can estimate contributes less to the total channel power. Let us now consider a scenario with imperfect CSI. Figure 12 shows the secrecy rate when the SNR is 12 dB and different values of $\alpha_{RP}$ are considered.

**Figure 13:**
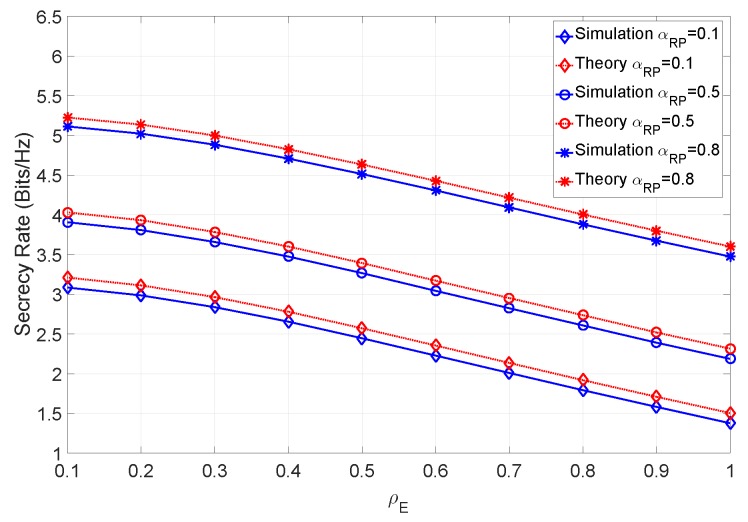
Secrecy rate of the system for various ray power ratios with $\beta_{N}=100$ and $\beta_{M}=10$ at 12 dB SNR.

The unknown multipath component introduces a permanent error in the eavesdropper, which accounts for the higher secrecy rate at $\rho_{E}=1$, similar to the mismatch error. In Figure 13, we have introduced the mismatch error to the previous scenario.

We verify that the mismatch error leads to an overall increased secrecy rate at all power ratios, since by varying this ratio, only the eavesdropper’s channel estimate and the corresponding capacity is affected.

## 4. Conclusions

In this paper, we proposed a physical security level against eavesdroppers which is based on MIMO-SVD schemes along with SC-FDE techniques. The security potential was studied, and it was shown that the secrecy rate can increase sharply as the distance between eavesdropper and transmitter or receiver increases. It was also demonstrated that in LOS scenarios, the secrecy rate increased with the multipath component’s power. Therefore, MIMO-SVD schemes combined with SC-FDE tecnhiques can be an efficient option for highly secure MIMO communications.
